# An Optimal Free Energy Dissipation Strategy of the MinCDE Oscillator in Regulating Symmetric Bacterial Cell Division

**DOI:** 10.1371/journal.pcbi.1004351

**Published:** 2015-08-28

**Authors:** Liping Xiong, Ganhui Lan

**Affiliations:** Department of Physics, George Washington University, Washington, D.C., United States of America; Universität des Saarlandes, Germany, GERMANY

## Abstract

Sustained molecular oscillations are ubiquitous in biology. The obtained oscillatory patterns provide vital functions as timekeepers, pacemakers and spacemarkers. Models based on control theory have been introduced to explain how specific oscillatory behaviors stem from protein interaction feedbacks, whereas the energy dissipation through the oscillating processes and its role in the regulatory function remain unexplored. Here we developed a general framework to assess an oscillator’s regulation performance at different dissipation levels. Using the *Escherichia coli* MinCDE oscillator as a model system, we showed that a sufficient amount of energy dissipation is needed to switch on the oscillation, which is tightly coupled to the system’s regulatory performance. Once the dissipation level is beyond this threshold, unlike stationary regulators’ monotonic performance-to-cost relation, excess dissipation at certain steps in the oscillating process damages the oscillator’s regulatory performance. We further discovered that the chemical free energy from ATP hydrolysis has to be strategically assigned to the MinE-aided MinD release and the MinD immobilization steps for optimal performance, and a higher energy budget improves the robustness of the oscillator. These results unfold a novel mode by which living systems trade energy for regulatory function.

## Author Summary

This paper presents a unique dissipation mode of converting biochemical free energy in ATP to regulatory function through the MinCDE bio-oscillator that marks the mid-cell position for symmetric bacterial cell division. Through assessing the oscillator’s performance-to-cost relation, we demonstrate that some dissipation threshold needs to be satisfied to switch on the oscillation, but the oscillator’s performance can be damaged by excess free energy dissipation, which is distinct from the known monotonic tradeoff relation of stationary regulators. An optimal dissipation strategy has been unveiled: the ATP free energy must be precisely allocated to specific reaction steps for accurate mid-cell recognition, which also coincides with the dynamic requirements for robust oscillation to occur. These discoveries identify an optimizable operation scheme of free energy consumption in biological systems and provide deep insights into the evolution of dynamic regulatory networks.

## Introduction

Similar to man-made systems that commonly employ sustained oscillations to measure time and length, living organisms use molecular oscillators to process spatiotemporal information for regulation. For example, the periodic pole-to-pole oscillation of Min proteins in *Escherichia coli* designates the mid-cell position for symmetric cell division [1, 2]; the oscillatory spindle dynamics in *Caenorhabditis elegans* [3, 4] and human cells [5] help position and orient the spindle at proper division site along the cell body; the genetic [6, 7] and non-genetic [8, 9] circadian rhythm networks repeatedly reset the intracellular environment every 24 hours; the RhoA and stress-fiber mediated oscillation synchronizes and coordinates the development of cells in *Drosophila* embryo [10, 11]; the traveling and standing waves set the differentiation markers in the *zebrafish* segmentation process [12, 13]. These different types of oscillators all emerge from various promotive and inhibitive interactions between the involved protein molecules, and their vital functions have been investigated extensively. However, the costs to sustain those functions have been overlooked almost completely. In particular, the free energy costs to drive the highly dissipative oscillating process, and “exchange rate” at which living organisms trade free energy for the above oscillatory regulation functions, remain largely unexplored.

**Fig 1:**
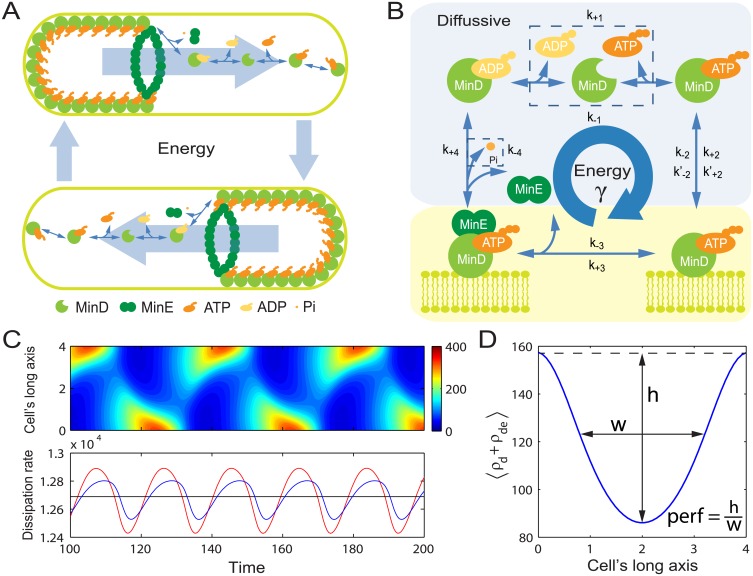
Schematic illustration of the MinCDE oscillator. (A) The ATP powered oscillation occurs between two poles. (B) Our model includes 4 microscopically reversible reaction steps as labeled: *1*) nucleotide exchange; *2*) MinD immobilization (spontaneously and cooperatively); *3*) MinE recruitment to membrane; *4*) MinE-aided MinD release (ATP hydrolysis). The rate *k*_+*i*_ is for clockwise reactions and *k*_−*i*_ for counter clockwise reactions (*i* = 1,2,3 & 4). These 4 reactions form a dissipative “futile” cycle. (C) Kymograph of the concentration of membrane bound MinD (upper panel) and the associated dissipation rates (lower panel) given by Eq (1) (blue) and Eq (16) (red), respectively. Their averages over one period collapse into the same black line as shown (Eq (2)). (D) The “V”-shaped time-averaged (over one period) concentration profile of membrane bound MinD along the cell’s long axis.

We address these questions by investigating the *E. coli* MinCDE oscillatory network for regulation of the cells’ symmetric division. This Min oscillator comprises three proteins: the division inhibitor MinC, the ATPase MinD, and the catalytic enzyme MinE. Experiments have shown that these Min molecules interact with each other under the mediation of ATP and the phospholipid membrane [14, 15]: the ATP-bound MinD cooperatively associates with the cell membrane [16, 17], where MinE and MinC are recruited [18, 19]; MinE enhances MinD’s ATPase activity, which releases MinD from membrane after ATP is hydrolyzed [15, 20]; meanwhile MinC reduces the stability of FtsZ polymers that construct the scaffold of the division ring [21], which in turn inhibits cell division at MinC-rich locations. Based on these protein-protein interaction logics, a molecular reaction-diffusion mechanism emerges: cytoplasmic MinD molecules associate with ATP and aggregate on the cell membrane at one of the two poles; MinE molecules chase and bind to this MinD colony, catalyze the ATP hydrolysis, and eventually destroy the MinD aggregation; the released MinD molecules will then diffuse to the other pole of the cell to start the aggregation process again. [22–27]. Such reaction-diffusion process gives rise to the spatiotemporal oscillation of Min molecules between two cell poles (Fig 1A, 1B and 1C). An emerged oscillatory pattern (S1 Movie) will then result, on time average, in a “V”-shaped concentration profile of MinC along the cell’s long axis with the minimum at the mid-cell [28] (Fig 1D).

It is known that any sustained biochemical oscillation is dissipative and requires continuous free energy input [29, 30]. In line with this general concept, the energy-bearing ATP is an essential element of the Min oscillator [14, 15]. Different from the existing theoretical models that emphasize the topological and dynamic properties of the Min protein network [22–26, 31], we construct a general analysis framework to quantify the dissipative nature of the oscillator as well as the biophysical role of ATP. Our results explicitly indicate that sufficient free energy dissipation is required to switch on the oscillator, but counterintuitively, we found that free energy input does not always promote the differentiation of the mid-cell from the cell poles (defined as the “performance” [31]; Fig 1D, also see Methods). Through a global optimization analysis, we further discovered that the best performance can only be achieved when most of the ATP hydrolysis energy is dissipated in the steps of the MinE-aided MinD release and the MinD immobilization, suggesting an optimal free energy dissipation strategy for *E. coli* under the pressure of natural selection. These discoveries set the Min oscillator apart from stationary regulators, such as the sensory adaption systems [32, 33] and the kinetic proofreading system [34–36], whose performance is monotonically improved by higher free energy dissipation. Thus, our results suggest different free energy conversion modes for stationary and oscillatory bio-regulators.

## Results

### The microscopically reversible reaction-diffusion network

To analyze the thermodynamic properties of the MinCDE oscillator, we constructed a detailed biochemical model (Fig 1A and 1B) based on the protein-protein interaction logics suggested in previous studies [25]. In this model, two regulatory motifs are involved for oscillation: positive auto-regulation (immobilized MinD cooperatively recruits more of itself) and negative feedback (the recruited MinE inhibits immobilized MinD *via* enhancing its ATPase activity). This category of interlinked positive and negative feedback loops are known to give rise to robust and tunable biological oscillations [37, 38]. Since MinC only contributes to ring-inhibition but not oscillation [2, 19], it is not explicitly included in this study of the Min oscillator.

A critical difference between our model and existing models is that we consider all reaction steps as “microscopically reversible” processes, which allows us to assess the free energy dissipation of individual steps through the forward and backward reaction fluxes [39]. These fluxes (*j*_±*i*_) can be expressed as:

$$\begin{array}{llll} j_{+1} & =k_{+1}\rho_{D:D}, & j_{-1} & =k_{-1}\rho_{D:T},\;\\ j_{+2} & =k_{+2}\rho_{D:T}, & j_{-2} & =k_{-2}\rho_{d},\;\\ j_{+2}^{\prime } & =k_{+2}^{\prime }\rho_{D:T}(\rho_{d}+\rho_{de}), & j_{-2}^{\prime } & =k_{-2}^{\prime }\rho_{d}(\rho_{d}+\rho_{de}),\;\\ j_{+3} & =k_{+3}\rho_{E}\rho_{d}, & j_{-3} & =k_{-3}\rho_{de},\;\\ j_{+4} & =k_{+4}\rho_{de}, & j_{-4} & =k_{-4}\rho_{D:D}\rho_{E},\; \end{array}$$

where *ρ*_*D*:*D*_, *ρ*_*D*:*T*_ & *ρ*_*E*_ are the concentrations of cytoplasmic MinD:ADP, MinD:ATP complexes and MinE dimers, respectively; *ρ*_*d*_ & *ρ*_*de*_ are the concentrations of MinD:ATP and MinE:MinD:ATP complexes on the membrane, respectively. The reaction index *i* (= 1,2,3 & 4) represents the reactions labeled in Fig 1B accordingly. It is worth noting that $j_{\pm 2}^{\prime }$ represent the cooperative recruitment of MinD:ATP to the cell membrane by existing membrane-associated MinDs (i.e. *d* & *de*).

For each reaction step, the net flux is *j*_*i*_ = *j*_+*i*_−*j*_−*i*_ (*i* = 1,2,3 & 4) (and $j_{2}^{\prime }=j_{+2}^{\prime }-j_{-2}^{\prime }$ for the cooperative recruitment). In consideration of the diffusion of protein molecules in the cell volume, the dynamics of the system can therefore be described by a set of reaction-diffusion equations (see Methods for details), and if detailed balance is satisfied, the parameter

$$\gamma \equiv \frac{k_{+1}k_{+2}k_{+3}k_{+4}}{k_{-1}k_{-2}k_{-3}k_{-4}}$$

shall be unity; otherwise, the system is out of equilibrium and the chemical free energy from ATP hydrolysis is constantly dissipated.

### Free energy dissipation of the reaction-diffusion system

The developed microscopically reversible reaction network allows us to assess the dissipation level of the oscillator. However, although the physics and methodology for evaluating free energy dissipation (or entropy production) of chemical reaction systems with stationary solutions has been nicely reviewed [29, 39], to our knowledge, dissipation of reaction-diffusion systems has yet to be broadly investigated [40]. A seemingly straightforward way to analyze such spatiotemporally varying systems is to compute and integrate the unbalanced fluxes of all reaction steps, as well as the diffusion processes (see Methods for details). Here, we obtain the total free energy dissipation rate of the reaction-diffusion system in a more elucidating way.

For any open biochemical reaction system in a steady or sustained oscillatory state, its average free energy dissipation rate is equal to the average consumption rate of the chemical free energy embedded in the environmental fuel molecules [41]. In our case of the Min oscillator, ATP is the energy source continuously supplied from the cytoplasm. The oscillator uptakes ATP molecules and excretes the hydrolysis products (ADP and inorganic phosphate, Pi) in the nucleotide exchange step (*k*_±1_) and the MinE-aided MinD release step (*k*_±4_, which is also the ATP hydrolysis step), respectively. Therefore, the chemical free energy supplied from ATP should directly be the free energy consumed by the oscillator, which is ln *γ* *per* ATP (in unit of *k*_*B*_ *T*) [41]. It is worth noting that this free energy depends on the cytoplasmic concentrations of ATP, ADP and Pi, and is different from the standard free energy change of ATP hydrolysis [42]. Hence, the continuous free energy dissipation rate is:

$$\sigma_{\text{ATP}}\left(t\right)=\int_{V}dxj_{1}\left(x,t\right)\ln\gamma \equiv J_{1}\left(t\right)\ln\gamma ,$$ (1)

where the integral is taken over the cell volume, and the integrated flux *J*_1_(*t*) is the number of MinDs transiting from MinD:ADP to MinD:ATP per unit time. For a stable stationary system, $J_{1}=J_{2}+J_{2}^{\prime }=J_{3}=J_{4}\equiv J$ is invariant over time; whereas for a sustained oscillatory system, the flux-balance relation holds only for time average over the oscillation period (*P*): $\left\langle J_{1}\rangle \equiv P^{-1}\int_{t_{0}}^{t_{0}+P}dtJ_{1}(t)=\langle J_{2}+J_{2}^{\prime }\rangle =\langle J_{3}\rangle =\langle J_{4}\rangle \equiv \langle J\right\rangle$.

From an energetic point of view, *σ*_ATP_ represents the chemical free energy “deposit” rate through uptaking ATP molecules from the cell’s cytoplasm. One should be aware that under sustained oscillating condition, the time averaged *σ*_ATP_ over each period is equal to the time average of the instantaneous dissipation rate *σ*_tot_ (Fig 1C), i.e.

$$\left\langle \sigma_{\text{ATP}}\right\rangle =\left\langle \sigma_{\text{tot}}\right\rangle =\left\langle J\right\rangle \ln\gamma \equiv \left\langle \sigma \right\rangle .$$ (2)

We use this average dissipation rate for sustained oscillation in our analysis. Also, to analyze the system’s behavior within the same framework, we use ⟨*σ*⟩ to denote the dissipation rate for non-oscillatory states whose average value is equal to the instantaneous value. It is straightforward from Eq (2) that the equilibrium situation (*γ* = 1) is non-dissipative.

### Sufficient energy is required to switch on the oscillator

Eq (2) provides a convenient way to compute free energy dissipation. We first investigate how the dissipation rate ⟨*σ*⟩ depends on the non-equilibrium parameter *γ* by tuning the backward nucleotide exchange step (MinD:ATP → MinD:ADP, *k*_−1_), while keeping other reaction rates fixed (see Methods).

**Fig 2:**
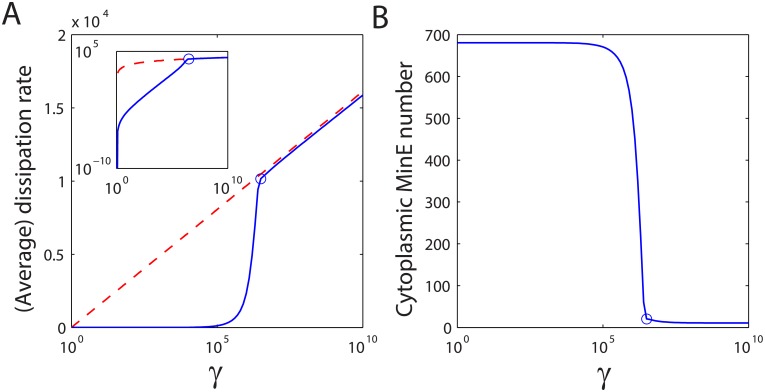
Dissipated free energy (A) and the number of cytoplasmic MinE (B) as functions of *γ* by changing *k*_−1_. The inset shows the same dissipation free energy, but in logarithmic scale. Oscillation occurs when *γ* > 2.8 × 10^6^ (circles). The dissipation rate is very close to the theoretical limit obtained from kinetic analysis (Eq (3), red dashed line) in the oscillatory regime where almost all the MinE dimers are bound on the membrane. Parameters used in the simulation are: *k*_−2_ = 0.01 *s* ^−1^, $k_{-2}^{\prime }=0.001\;\mu m^{2}s^{-1}$, *k*_−3_ = 0.04 *s* ^−1^ and *k*_−4_ = 10^−5^ *μm* ^4^ *s* ^−1^. Other parameters are given in the main text.

The system exhibits two distinct regimes for the ⟨*σ*⟩ − *γ* dependence: for small *γ*, ⟨*σ*⟩ initially increases slowly with ln *γ*; when *γ* is further increased, ⟨*σ*⟩ increases dramatically with ln *γ* and then settles to a logarithmic regime where ⟨*σ*⟩ ∝ ln *γ* (Fig 2A; S1 and S2 Figs). The threshold between the two regimes is the same transition point for oscillation to occur (circle in Fig 2A), and the Min system exhibits only the regulatory function in the oscillatory regime (in the non-oscillatory stable regime, MinD is distributed uniformly on the membrane or slightly higher in the middle due to the cell-end effect as shown in S6 Fig). It is worth noting that the logarithmic dependence has been also observed in the stationary sensory adaptation systems when the net fluxes ⟨*J*⟩ reach their saturated levels [32, 33]. Our results suggest that this category of logarithmic relation between the non-equilibrium parameter and the associated free energy dissipation may be a universal qualitative indicator of a biochemical network providing its regulatory function, regardless of it being intrinsically stationary or oscillatory.

To investigate further how this logarithmic regime coincides with the oscillatory behavior of the system, we analyze the spatiotemporal patterns of Min protein molecules while the system becomes more dissipative. We find that the MinE dimers remain mostly in cytoplasm until the same dissipative “threshold” is reached; beyond this “threshold”, most MinEs are confined onto the membrane (Fig 2B). Due to the effective inhibitive role of MinE on MinD:ATP being associated with the membrane, oscillation can occur only at low cytoplasmic MinE molecule numbers. Therefore, when oscillation is switched on, the average flux is rate-limited by the MinE number: ⟨*J*⟩_osc_ ≈ ⟨*J*⟩_max_ = *k*_+4_ *N*_*E*_, and the free energy dissipation is then (Fig 2A, dashed line):

$${\left\langle \sigma \right\rangle }_{\text{osc}}\approx k_{+4}N_{E}\ln\gamma .$$ (3)

Similar threshold and logarithmic dependence of free energy dissipation on *γ* can also be observed by constraining other reverse reaction rates from their equilibrium values (S3 and S4 Figs), and the dissipation rate can still be approximated by Eq (3) in the oscillatory/regulatory regime (see S1 Text for details).

### Excess free energy dissipation may repress the oscillatory spatial regulation

The threshold to switch on the oscillator (Fig 2) indicates that oscillation occurs only when the dissipated free energy is large enough to sequestrate MinE from cytoplasm. Because the nucleotide exchange and MinE-aided MinD release steps are the two material exchange steps between the oscillator and the intracellular environment, we systematically investigate how the oscillator’s regulatory performance depends on these two interfaces.

**Fig 3:**
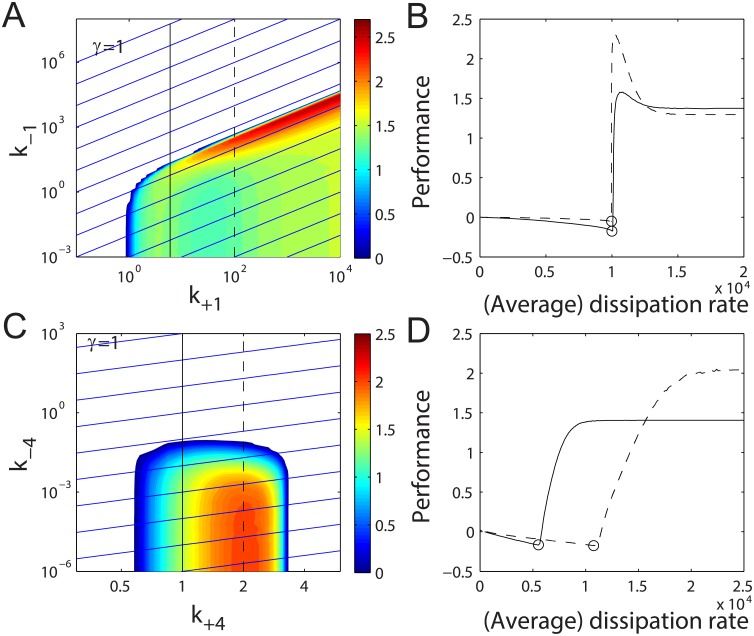
Performance as a function of reaction rates and free energy dissipation. (A, C) Contour plots of performance (colored region) and *γ* (blue lines) in (*k*_−1_, *k*_+1_) and (*k*_−4_, *k*_+4_) spaces, respectively. *γ* increases incrementally from 1 to higher values and the adjacent blue lines are separated by ten-fold. (B) The performance-to-cost relations are along the solid (*k*_+1_ = 6 *s* ^−1^) and the dashed (*k*_+1_ = 100 *s* ^−1^) trajectories in (A). (D) The performance-to-cost relations are along the solid (*k*_+4_ = 1 *s* ^−1^) and the dashed (*k*_+4_ = 2 *s* ^−1^) trajectories in (C). The circles mark the bifurcation points of each trajectory. For (A, B), *k*_−4_ = 10^−5^ *μm* ^4^ *s* ^−1^, and for (C, D), *k*_−1_ = 0.6 *s* ^−1^. For both cases, *k*_−2_ = 0.01 *s* ^−1^, $k_{-2}^{\prime }=0.001\;\mu m^{2}s^{-1}$ and *k*_−3_ = 0.04 *s* ^−1^. Other parameters are given in the main text.

Fig 3A and 3C show the performance analysis in the (*k*_+1_, *k*_−1_) and (*k*_+4_, *k*_−4_) spaces, respectively. The sharp bifurcation boundaries separate the oscillatory (colored) and stationary (white) regions: for the nucleotide exchange step, our result indicates that a fast ATP replacement rate and a large *γ* are both necessary for the oscillator to operate. In contrast, for the MinE-aided MinD release step, the system oscillates only when the releasing rate (*k*_+4_) remains within a moderate range while the rebinding rate (*k*_−4_) stays low. Despite the quantitative difference in the parameter scales, the regulatory performance is positive only in the oscillatory regions, and a minimum free energy dissipation needs to be satisfied for sustained Min oscillation for both material exchange interfaces.

Once the energetic threshold is met, the system reacts differently to the tuning of these two interfaces. When its dissipative behavior is tuned through the nucleotide exchange step (*k*_±1_), the system achieves its best performance in the region adjacent to the bifurcation boundary (red region in Fig 3A). A more explicit representation is given in Fig 3B: the solid and dashed lines correspond to the solid and dashed lines in Fig 3A, along which the regulatory performance and energetic costs are quantified. In both cases, the system’s performance first increases from *zero* to a finite peak value, and then decreases to a lower level (see panels A & B in S5 Fig for the membrane-associated MinD profiles). The negative performance before the bifurcation is due to the cell-end effect (S6 Fig).

On the other hand, when the system is tuned through the MinE-aided MinD releasing step (*k*_±4_), the high performance region is buried deeply in the oscillatory phase, and the performance exhibits simple monotonic dependence on free energy dissipation (Fig 3C and 3D and panels C & D in S5 Fig): at any given *k*_+4_, a higher free energy dissipation (i.e. smaller *k*_−4_) always leads to a better performance in mid-cell recognition. Interestingly, if we hold *k*_−4_ constant and vary *k*_+4_ (along a horizontal line in the performance map), the performance to dissipation relation would be non-monotonic with the best performance being achieved at moderate *k*_+4_ values.

These qualitatively distinct performance-to-cost relations indicate that not only different transition steps have different roles in the regulation process, but also the forward and backward reactions in the same step have different influences on the regulatory function. Moreover, the existence of an “optimal” operating region is distinct from other known stationary regulatory systems (e.g. sensory adaptation and kinetic proofreading systems) whose performance always improves monotonically with increasing free energy dissipation [32–36], suggesting that, in addition to direct free energy consumption, more complex dynamic requirements have to be satisfied for the Min oscillator to assume the role as an efficient mid-cell marker.

### Optimal dissipation strategy for high performance under limited energy budget

**Fig 4:**
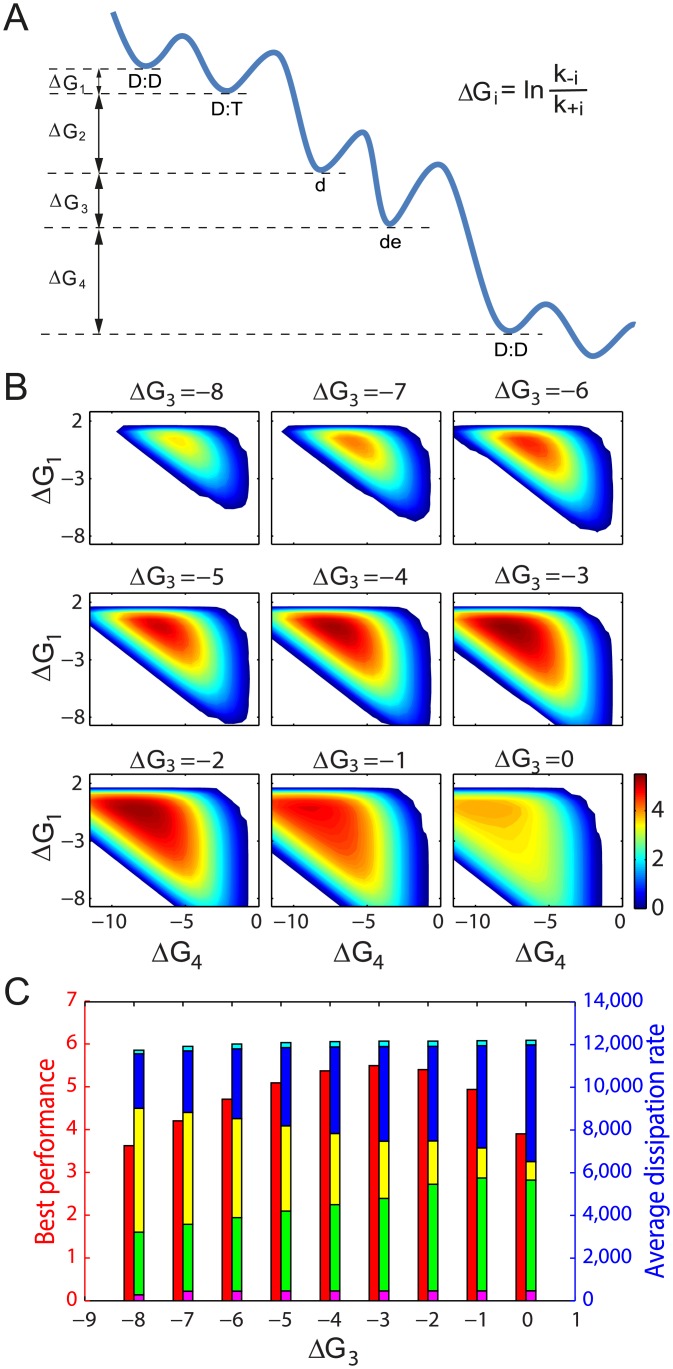
Dissipation strategy with fixed physiological level free energy budget. (A) An illustration of free energy landscape through the reaction “futile” cycle. Δ*G*_*i*_ = ln(*k*_−*i*_/*k*_+*i*_) is the “standard” free energy change of the *i* ^*th*^ reaction. The total free energy dissipation of each MinD ATPase cycle (whose absolute value is equal to the free energy released from one ATP) is $\Delta G=\sum_{i=1}^{4}\Delta G_{i}=-\text{ln}\;\gamma$. (B) Contour plots of the performance in (Δ*G*_1_,Δ*G*_4_) space at different Δ*G*_3_. Δ*G* is fixed at −18 *k*_*B*_ *T*. (C) The highest performance at different Δ*G*_3_ is identified from B and plotted as red bar. The corresponding dissipation rates of the reactions 1 (magenta), 2 (green), 3 (yellow), 4 (blue) and the diffusion process (cyan) are plotted as labeled color bars.

The results shown in Fig 3 suggest that the regulatory performance of the Min oscillator does not depend simply on how much free energy is dissipated, but indeed on how exactly the free energy is dissipated through the individual steps in the reaction-diffusion process. To investigate the *E. coli* cell’s dissipation strategy in mid-cell determination, we performed a systematic analysis of the Min oscillator’s performance by changing all the reverse reaction rates (*k*_−*i*_, *i* = 1 ∼ 4) while keeping *γ* fixed at the physiological level (i.e. ln *γ* is maintained at around 18 *k*_*B*_ *T* to match the chemical free energy released from hydrolyzing each ATP molecule in *E. coli*[42]). This is equivalent to assigning different free energy consumption to different reaction steps while keeping the total free energy budget constant (given in Eq (3); see Fig 4A for illustration).

Fig 4B summarizes the results of our *in silico* experiments. In this figure, we use Δ*G*_*i*_ = ln *k*_−*i*_/*k*_+*i*_ to denote the standard free energy difference through the *i*th reaction step. It is worth special attention that the “standard” condition used here is slightly different from the conventional standard condition: since ATP, ADP and Pi are not explicitly included as reactants/products in our model but are implicitly embedded in the reaction rates *k*_±*i*_, we set our “standard” condition to be at unit concentration of Min molecules and at physiological levels of ATP, ADP and Pi. Since the total $\Delta G=\sum_{i=1}^{4}\Delta G_{i}=-\text{ln}\;\gamma$ *per* ATP is constant, only three out of four of the reactions are independent. In each elementary panel of Fig 4B, we present the performance contour plots in the (Δ*G*_1_, Δ*G*_4_) space at different Δ*G*_3_ values. The best performance point in each contour plot is identified and plotted in Fig 4C, where the red bars show the best performance scores at given Δ*G*_3_ levels, and the partitioned color bars indicate the calculated free energy dissipation rates for each of the individual reactions (*magenta*, *green*, *yellow* & *blue* for *nucleotide exchange*, *MinD immobilization*, *MinE recruitment* & *MinE-aided MinD release*, respectively) and the *diffusion process* (*cyan*) to achieve the best performance. The total heights of the partitioned color bars directly represent the total free energy dissipation rates, which are nearly constant (close to *k*_+4_ *N*_*E*_ ln *γ* = 12,600 *k*_*B*_ *Ts* ^−1^ ∼ 700 ATPs *per* second).

These results provide a full spectrum picture of how energy is used to promote the Min oscillator’s performance. Firstly, oscillation occurs only within certain dissipation-strategy regions (colored in Fig 4B). A “bad” strategy can eliminate the oscillations, therefore having no regulatory function even with abundant energy source. Secondly, under the global optimal scenario (Δ*G*_3_ = −3 *k*_*B*_ *T* in Fig 4C, where the performance score is globally the highest), the largest amount of free energy (4,440.9 *k*_*B*_ *Ts* ^−1^, 36.5% of ⟨*σ*⟩_osc_) is dissipated in the MinE-aided MinD release step, where ATP is hydrolyzed and Pi is released; a similar amount of free energy (4,332.5 *k*_*B*_ *Ts* ^−1^, 35.6% of ⟨*σ*⟩_osc_) is dissipated to ensure efficient MinD immobilization onto the cell membrane; a significant amount of free energy (2,681.4 *k*_*B*_ *Ts* ^−1^, 22% of ⟨*σ*⟩_osc_) is dissipated to recruit MinE to the membrane; and the least free energy (460.4 *k*_*B*_ *Ts* ^−1^, 3.8% of ⟨*σ*⟩_osc_) is used for nucleotide exchange (the rest 2.1% is dissipated in diffusion). From a structural point of view, it is known that binding of ATP helps the MinD molecule to rearrange its structure for a higher affinity to phospholipid and to MinE [43–45]. Therefore, the large amount of free energy dissipated in the membrane and the MinE involved steps might suggest that, under the pressure of natural selection, *E. coli* cells may have evolved to use the energy bearing ATP molecules fully to coordinate with the necessary structural changes for optimal operation. This theoretically-obtained optimal partition also clearly indicates that different reaction steps play different roles in converting free energy for the oscillatory functions.

We want to point out that the above dissipation partitions, as well as the color bars in Fig 4C, are calculated using the time averaged dissipation rates in individual reaction steps ⟨*σ*_*i*_⟩:

$$\left\langle \sigma_{i}\right\rangle =\frac{1}{P}\int_{P}dt\sigma_{i}\left(t\right)=\frac{1}{P}\int_{P}dt\int_{x}dxj_{i}\left(x,t\right)\ln\frac{j_{+i}\left(x,t\right)}{j_{-i}\left(x,t\right)}.$$

Taking reaction 1 for example,

$$\left\langle \sigma_{1}\right\rangle =-\left\langle J\right\rangle \Delta G_{1}+\frac{1}{P}\int_{P}dt\int_{V}dxj_{1}\left(x,t\right)\ln\frac{\rho_{D:D}\left(x,t\right)}{\rho_{D:T}\left(x,t\right)},$$

which is different from Δ*G*_1_ = ln(*k*_−1_/*k*_+1_), which is the standard free energy difference between reactants and products under cytoplasmic ATP, ADP and Pi concentrations. Δ*G*_*i*_ is useful for understanding the reaction landscape of the system as illustrated in Fig 4A, but ⟨*σ*_*i*_⟩ represents the true dissipation at particular reaction steps (colored bars in Fig 4C). It is also worth noting that Eq (2) leads to ⟨*σ*⟩ = −⟨*J*⟩Δ*G* (i.e. the dissipated free energy is equal to the chemical free energy consumed through ATP hydrolysis).

### A higher energy budget leads to higher robustness of the oscillator

**Fig 5:**
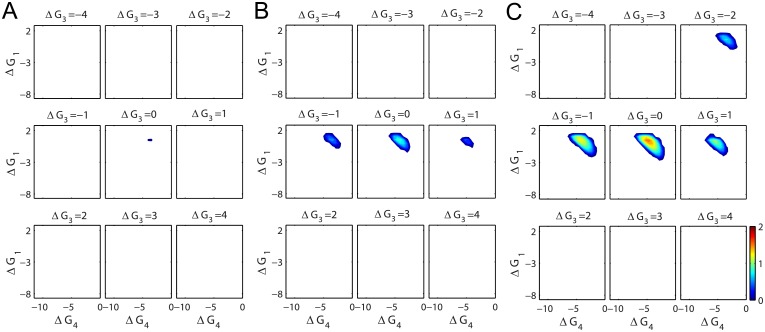
Contour plots of the performance in (Δ*G*_1_, Δ*G*_4_) space at different Δ*G*_3_. The total free energy dissipation in each MinD ATPase cycle (i.e., Δ*G* = −ln *γ*) is fixed at −6 *k*_*B*_ *T* (A), −7 *k*_*B*_ *T* (B) and −8 *k*_*B*_ *T* (C) respectively. At ln *γ* = 6 *k*_*B*_ *T* (A), the system needs to finely tune its parameters (dissipation strategy) into a small region (colored) to oscillate. Outside this region (white), no oscillation can occur. With increasing amounts of energy source (B, C), the oscillatory region expands, indicating a more robust oscillation.

**Fig 6:**
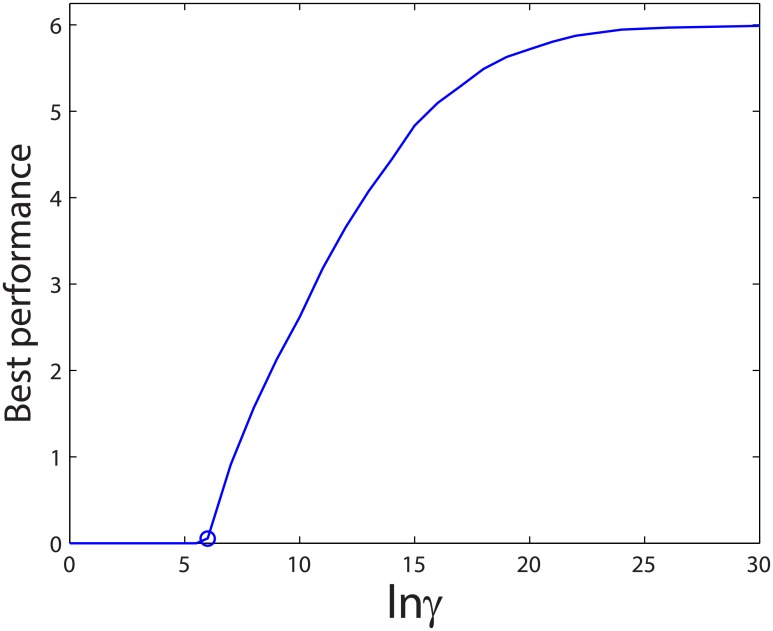
The best performance score as a function of total energy budget. As ln *γ* increases, the highest possible performance score also increases until a saturation level is reached. The Min oscillator exhibits a global minimum requirement for the free energy dissipation, which is around 6*k*_*B*_ *T* as marked by the circle in the curve.

Maintaining the above two dynamic conditions requires sufficient energy input to break symmetry in the proteins’ concentration distribution between the two poles. Fig 2A shows a minimum *γ* value obtained from tuning the nucleotide exchange step. We extended our analysis to a broader biochemical space to include all reverse reaction steps, and we further applied the global optimization method to different *γ* values (i.e. total energy budgets for dissipation). Our results show that, the Min oscillation can never be switched on if the total energy budget is too low, no matter how the system is optimized (Figs 5 and 6). This minimum value of ln *γ* is around 6 *k*_*B*_ *T*. By comparing the operation phase plots at different *γ* values (Figs 4B and 5), we clearly show that the higher the total energy budget, the bigger the area in which oscillation could occur, implying higher robustness of the oscillator. Thus with an adequate energy budget, the organism has more freedom to arrange its internal environment while maintaining the vital oscillatory function.

Fig 6 summarizes the global optimal performance with increasing *γ*. By carefully tuning the Min pathway for optimization, a higher energy budget can eventually lead to a better global optimal performance until a saturation level is reached. This result indicates that the total energy budget is important for the highest achievable differentiation between the mid-cell and the pole regions, but the detailed dissipative strategy plays a more direct role in controlling the actual performance that the system can deliver.

## Discussion

Living organisms are all dissipative and the dissipated free energy is used to perform mechanical work, facilitate bio-synthesis/degradation processes, and process regulatory information. However, although it is intuitive to connect energy to mechanical work and to bio-mass turnover, a bigger challenge is the quantitative understanding of the free energy conversion mechanism through biological regulation processes. This difficulty is mainly because the free energy consumption in biological regulation is hardly accessible at the molecular level, and meanwhile the embedded information processing is difficult to quantify and evaluate. To date, only two energy-regulation conversion schemes have been quantitatively identified: the KP scheme and the ESA tradeoff scheme. The KP scheme was proposed by Hopfield for the *K*inetic-*P*roofreading processes whose regulatory accuracy can be enhanced through every cycle of enzymatic checking [34–36]. In this scheme, the dissipated free energy effectively lowers the energy of an already stable state. On the other hand, it was recently discovered the ESA scheme in the sensory adaptation systems that higher free energy consumption, which is the product of the *E*nergy dissipation rate and the inverse of the adaptation *S*peed, exponentially improves adaption *A*ccuracy. In those processes, energy is used to stabilize an originally unstable state [32, 33]. Both schemes are stationary and exhibit monotonic performance-to-cost relation.

In this work, we present a third energy-regulation conversion scheme that is unique for oscillatory regulators. Our results show that, although energy is necessary to sustain biochemical oscillation, the regulatory performance does not monotonically depend on the total free energy dissipated over the full reaction cycle. Instead, the oscillator’s performance largely depends on how the energy is partitioned and dissipated through individual catalytic steps. In particular, for the MinCDE system, an optimal dissipation strategy is to allocate most of the available free energy to the MinE-aided MinD release and the MinD immobilization steps (i.e. large *σ*_2_ & *σ*_4_), whereas the nucleotide exchange step has to be kept less dissipative (i.e. small *σ*_1_). This non-monotonic optimizable feature is distinct from the KP and ESA schemes.

Our results provide energetic insights into the question of what drives the MinCDE oscillation. As predicted from the reaction-diffusion mechanism, the sustained MinCDE oscillation would require an uninterrupted pole-to-pole passage of MinD molecules together with a strong confinement of MinE molecules to the cell membrane [31]. To guarantee the uninterrupted MinD passage, a MinD concentration gradient between the two cell poles has to be established and maintained during the fast diffusion process (∼ 0.5 seconds) with the following perspectives: from a mass transfer point of view, such a gradient requires a “source” at the previously MinD-occupied pole and a “sink” at the far end of the cell; from an energetic standpoint, the “source” and “sink” are maintained by the highly “dissipative” MinE-aided MinD release and the MinD immobilization steps, respectively. In particular, a large *σ*_4_ keeps the rebinding of MinD at low levels, providing a net inward flux of MinD from the membrane; whereas a large *σ*_2_ guarantees near-perfect absorption of MinD onto the membrane at the far end.

Once a gradient is established, a remaining task is to avoid MinD binding to other parts of the cell membrane other than the far end cell pole. To this end, two additional requirements have to be satisfied: 1) the nucleotide exchange step should have a relatively high reverse rate with low dissipation level (i.e. small *k*_+1_/*k*_−1_ ratio and small *σ*_1_) so that once MinD associates with ATP (high membrane affinity), it can easily exchange back to the ADP state (low membrane affinity); 2) the nucleotide exchange rates (*k*_±1_) have to be fast so that diffusion cannot flatten out the gradient profile before MinD is ready for membrane binding. The first condition reduces the chance of MinD binding to the membrane during its first passage down the gradient; whereas the second condition reduces the chance of “backfire” in which MinD diffuses back. These two requirements are evident in Fig 3A: the performance is higher near the bifurcation boundary than deep inside the oscillatory phase region; along the *γ* contour line (in this case, also the *k*_+1_/*k*_−1_ contour line) passing the bifurcation boundary, the larger the rates, the better the performance will be.

Sustained MinCDE oscillation also requires MinE dimers to be confined at the old pole and meanwhile the cytoplasmic MinE has to be maintained at a low level to allow MinD accumulation at the new pole. To satisfy this condition, the cooperative recruitment step, where MinE are recruited, acquires a significant amount of free energy (*σ*_3_) to create a strong recruiting trap for MinE. Therefore, the MinCDE oscillator uses the free energy in ATP to establish alternating gradients across the long axis of the cell, so that the MinD can diffuse to and cooperatively build up a colony at the far end of the cell before the MinE can chase and destroy it. Furthermore, the limited intracellular energy budget has to be partitioned wisely to balance the requirements from individual reaction steps. And our predicted optimal free energy dissipation strategy captures these dynamic logics, suggesting that energetic and dynamic requirements are deeply connected.

It is worth noting that our study is based on a deterministic picture of the problem. In a previous study by Rex et al., the stochastic effects from finite numbers of molecules have been investigated numerically using microscopically irreversible reactions [46]. They found that, at physiological levels of molecular numbers (> 2,000 molecules), the oscillatory behavior of the stochastic MinCDE system is close to those derived from the deterministic equations, and the noise of the oscillatory period and the averaged MinD concentration profile are at a relatively low level. These results encourage us to believe that our discoveries of how free energy is traded for better identifying the mid-cell position would still be valid in the context of a stochastic cellular environment. But it would require more detailed investigation to identify whether free energy dissipation could also contribute to reducing the noise arising from the finite numbers of molecules.

All existing theoretical models, for simplification, consider biological reactions as microscopically irreversible processes [22–27]. Although these models have successfully captured many qualitative and quantitative features of the studied biological systems, we show in this paper that the predicted behaviors of the MinCDE system from irreversible models do not converge to the optimal operating mode obtained from reversible analysis. In particular, our analysis (Fig 3A) explicitly shows that the MinCDE system can benefit from its reversibility and the reversible system is capable of achieving a much higher performance compared to the irreversible counterpart (note the maximum performance near the bifurcation boundary). These results indicate that the backward reaction does not just reduce the “net” forward reaction rate, but essentially enlarges the “volume” for the system’s dynamic trajectories to occupy, which in turn leads to richer dynamic behaviors.

Furthermore, the obtained performance-to-cost relation *via* reversible analysis implies that “energy efficiency” as well as the “operational robustness” might be important factors for living organisms to survive the pressure of natural selection, shedding light on the evolutionary principles of regulatory networks. Therefore, we believe that it is worthwhile to reexamine other biochemical systems using the reversible modeling framework introduced here for more comprehensive thermodynamic understanding.

## Methods

### MinCDE reaction-diffusion network

Given the net flux for each biochemical reaction step (*j*_*i* = 1,2,3 & 4_ and $j_{2}^{\prime }$), the dynamics of the reaction-diffusion system can be described as:

$$\frac{\partial \rho_{D:D}}{\partial t}=D_{D}\nabla^{2}\rho_{D:D}-j_{1},$$ (4)

$$\frac{\partial \rho_{D:T}}{\partial t}=D_{D}\nabla^{2}\rho_{D:T}+j_{1},$$ (5)

$$\frac{\partial \rho_{E}}{\partial t}=D_{E}\nabla^{2}\rho_{E},$$ (6)

where *D*_*D*_ & *D*_*E*_ are the diffusion constants of MinD:ADP and MinE in cytoplasm, respectively. The system’s boundary conditions are defined by the membrane-associated reactions on the cell membrane:

$$\frac{\partial \rho_{d}}{\partial t}=j_{2}+j_{2^{\prime }}-j_{3},$$ (7)

$$\frac{\partial \rho_{de}}{\partial t}=j_{3}-j_{4},$$ (8)

$$D_{D}n\cdot \nabla \rho_{D:D}=j_{4},$$ (9)

$$D_{D}n\cdot \nabla \rho_{D:T}=-j_{2}-j_{2}^{\prime },$$ (10)

$$D_{E}n\cdot \nabla \rho_{E}=-j_{3}+j_{4},$$ (11)

where **n** is the unit normal vector of the membrane pointing outward.

We want to point out that we adopt here the cooperative recruitment type of MinCDE oscillatory mechanism in which the diffusion on the cell membrane is considered unessential [25, 31, 47]. Similar analyses can be carried out for the other non-linear aggregation type of mechanism where 2-dimensional diffusion on the membrane plays an important role [24, 26], or for the recent comprehensive study where the two mechanisms and the direct binding of MinE to the membrane [18, 48, 49] are taken into account [27]. These would be beyond the scope of this paper, but we expect that qualitatively similar conclusions would be reached.

### Model parameters and characteristics

Using COMSOL Multiphysics 4.3, we simulate the *E. coli* cell as a cylinder with radius *R* = 0.5 *μm* and length *L* = 4 *μm*. The diffusion constants are set to be *D*_*D*_ = 16 *μm* ^2^ *s* ^−1^ and *D*_*E*_ = 10 *μm* ^2^ *s* ^−1^[50]. The total numbers of MinD particles and MinE dimers are fixed at *N*_*D*_ = 2,000 and *N*_*E*_ = 700, respectively [51]. Unless stated otherwise, the forward reaction rates are chosen from experimental measurements and previously theoretical studies: *k*_+1_ = 6 *s* ^−1^, *k*_+2_ = 0.1 *μms* ^−1^, $k_{+2}^{\prime }=0.01\;\mu m^{3}s^{-1}$, *k*_+3_ = 0.4 *μm* ^3^ *s* ^−1^ and *k*_+4_ = 1 *s* ^−1^[17, 18, 25, 27, 31]. The backward rates, on the other hand, are tuned to alter the system from equilibrium to non-equilibrium and are specified in the corresponding sections of the paper. In addition, although many experiments have confirmed that the binding process of MinD to the membrane is cooperative, yet lipid specific [17, 18], the detailed mechanism is unclear. In this paper, we adopt a simple catalytic view that treats the spontaneous (*j*_±2_) and the cooperative attachment ($j_{\pm 2}^{\prime }$) with the same free energy change. Therefore, $k_{+2}/k_{-2}=k_{+2}^{\prime }/k_{-2}^{\prime }$ is satisfied in our minimal simulation setup. Using these parameters, the oscillation period is around 40s as long as *k*_−4_ stays small as shown in S7 Fig, which is in agreement with experimental observations [2, 52]. S1 Movie shows an example of the oscillatory dynamics.

### Oscillation detection and performance quantification

Pole-to-pole oscillation is tightly coupled with the inequality of Min protein concentrations along the cell’s long axis. In our performance studies, we use an automated method to first detect the oscillatory dynamics: we collect the species’ concentrations over long time (for example, MinD concentration at one of the cell poles), and evaluate the variances of the time course data in steady state. The system is regarded to undergo oscillatory dynamics if the variance is non-zero.

Based on the time-averaged concentration profile along a cell’s long axis, we apply Halatek and Frey’s definition for the regulatory performance [31]: let *h* and *w*, respectively, be the normalized depth and width of the valley of the concentration profile of the membrane-bound MinD and MinD:MinE complex; then the performance is defined as *h*/*w*. (Fig 1D shows a particular example which has one extremum. A more detailed and general definition/illustration can be found in S8 Fig)

During oscillation, due to the canalized MinD transfer, MinD molecules periodically switch their occupancy at two cell poles. This results in a profile with lower MinD concentration at the mid-cell region. Therefore, the oscillation and the system’s positive performance are tightly coupled. A narrower and deeper valley at the mid-cell is considered superior for correct symmetric cell division, and is quantified by a higher performance score. We use such scores to demonstrate the relation between the regulatory performance and the associated energetic cost.

### Free energy dissipations of individual reaction steps and diffusion

We use the imbalanced fluxes in the reaction-diffusion process to calculate the free energy dissipation rate.

**Free energy dissipations in individual reaction steps**. For a particular chemical reaction *i*, if the forward and backward flux densities at a spatial position **x** are *j*_+*i*_(**x**, *t*) and *j*_−*i*_(**x**, *t*) respectively, the dissipation rate density is [39]:

$$\begin{array}{ll} \sigma_{i}(x,t) & =[j_{+i}(x,t)-j_{-i}(x,t)]\text{ln}\frac{j_{+i}(x,t)}{j_{-i}(x,t)}\\ & =j_{i}(x,t)\text{ln}\frac{j_{+i}(x,t)}{j_{-i}(x,t)}. \end{array}$$ (12)

For notational simplicity, the spontaneous and cooperative MinD attachments are denoted as one reaction here, i.e., $\sigma_{2}=(j_{2}+j_{2}^{\prime })\;\text{ln}(j_{+2}/j_{-2})$. The total dissipation rate of all the reactions is therefore:

$$\sigma_{\text{tot}}^{\text{react}}\left(t\right)=\int_{V}dx\sigma_{1}\left(x,t\right)+\int_{S}dx\sum_{i=2}^{4}\sigma_{i}\left(x,t\right),$$

where the subscripts _*V*_ and _*S*_ denote the cell volume and cell surface integrals, respectively.

**Free energy dissipations in diffusion**. The dissipation of the diffusion process is less obvious. We model the diffusion process as a number of particles performing random walks on a uniform 3-dimensional lattice space with grid size *dxdydz*. Each molecule on one node (**x** ≡ (*x*, *y*, *z*)) can jump to its six neighboring nodes with rate *a*. Taking two neighbors along *x* direction for example, the forward and backward fluxes are

$$j_{+x}\left(x\right)=j\left(x\to x+dx,y,z\right)=a\rho \left(x,y,z\right)dx$$

$$j_{-x}\left(x\right)=j\left(x+dx\to x,y,z\right)=a\rho \left(x+dx,y,z\right)dx$$

This leads to the well-known equality for the net flux:

$$j_{x}\left(x\right)=\lim_{dx\to 0}a\left[\rho \left(x,y,z\right)-\rho \left(x+dx,y,z\right)\right]dx=-D\frac{\partial \rho (x)}{\partial x}$$

and the diffusion constant is defined as *D* = lim_*dx* → 0_ *a*(*dx*)^2^. The dissipation rate between these two neighbors is:

$$\lim_{dx\to 0}dydzj_{x}\left(x\right)\ln\frac{j_{+x}\left(x\right)}{j_{-x}\left(x\right)}=dxdydz\frac{j_{x}^{2}\left(x\right)}{D\rho (x)},$$ (13)

where *j*_*x*_(**x**) is the *x* component of the net diffusion flux **j**(**x**) at location **x**. Hence the dissipation rate density of diffusion is

$$\sigma^{\text{diff}}\left(x,t\right)=\frac{j_{x}^{2}+j_{y}^{2}+j_{z}^{2}}{D\rho (x,t)}=\frac{{\left|j\left(x,t\right)\right|}^{2}}{D\rho (x,t)}.$$ (14)

This final result in terms of the flux vector is independent of coordinates. Because we disregard the diffusion on cell membrane, the total dissipation rate of the diffusion processes of the three diffusive cytoplasmic molecules can be written as:

$$\sigma_{\text{tot}}^{\text{diff}}\left(t\right)=\int_{V}dx\left[\sigma_{D:T}^{\text{diff}}\left(x,t\right)+\sigma_{D:D}^{\text{diff}}\left(x,t\right)+\sigma_{E}^{\text{diff}}\left(x,t\right)\right],$$ (15)

where the integral is taken over the cell volume. We find that the dissipation of the diffusion process is small compared to the reactions (see Fig 4C).

Combining reaction and diffusion, the total dissipation rate of the system can be calculated by summing these two parts:

$$\sigma_{\text{tot}}\left(t\right)=\sigma_{\text{tot}}^{\text{react}}\left(t\right)+\sigma_{\text{tot}}^{\text{diff}}\left(t\right).$$ (16)

Please note that the instantaneous dissipation rates calculated from Eqs (1) and (16) are different for oscillatory systems. However, the total dissipated free energy over each period is the same from these two analysis methods (as shown in Eq (2) and Fig 1C).

### Numerical simulation

We use COMSOL Multiphysics 4.3 to solve the partial differential equations. The cell is set up as a cylindrical volume with axial symmetry, which leaves us with a mathematical 2-dimensional model. The maximum grid size is set to be 0.1 *μm*. The entire system is then simulated using two physics modules provided by COMSOL: the “Transport of Diluted Species” module is used to simulate the reaction-diffusion process in cytoplasm, and the “Boundary ODEs and DAEs” module is used to account for the reactions on the cell membrane. These two modules are coupled: the membrane reactions serve as the boundary condition for the cytoplasmic reaction-diffusion process. The deterministic equations have an unstable solution with a uniform distribution of all species, so to break this unstable symmetry solution we used a step function as our initial condition for the MinD:ADP concentration in cytoplasm, while making MinD:ADP and MinE homogeneously distributed in the cytoplasm. All the simulations are run for a time long enough to cover at least 10 full periods of sustained oscillation with stable amplitude, and data are collected for these stable oscillations.

## Supporting Information

S1 FigMolecular numbers (A-E) and total flux (F) as functions of *γ* which is changed *via* changing *k*_−1_.Both cooperative-attachment (solid curves) and non-cooperative models (dashed curves) show threshold behaviors, but only the cooperative model is able to oscillate. The circles mark the bifurcation point. For the cooperative model, parameters are chosen to be: *k*_−2_ = 0.01 *s* ^−1^, $k_{-2}^{\prime }=0.001\;\mu m^{2}s^{-1}$, *k*_−3_ = 0.04 *s* ^−1^ and *k*_−4_ = 10^−5^ *μm* ^4^ *s* ^−1^. Other parameters are given in the main text. The non-cooperative model (without $k_{+2}^{\prime }$ and $k_{-2}^{\prime }$) has the same parameter values as the cooperative model except that *k*_+2_ = 1 *μms* ^−1^ and *k*_−2_ = 0.1 *s* ^−1^ to keep the molecular numbers and fluxes of the two models comparable.(EPS)

S2 FigDissipation rate (A), flux (B) and cytoplasmic MinE number (C) as functions of *γ* which is changed *via* changing *k*_−1_.Oscillation occurs when *γ* enters the logarithmic region and *k*_+1_ > 0.89 *s* ^−1^ (above black curve). (A) The dissipation rate in the oscillation region is very close to the theoretical limit (red dashed line). (B) The average flux saturates at large *γ*. (C) Almost all the MinE dimers are bound on the membrane in the oscillation region (blue curve). The circles mark the bifurcation point. The parameters are chosen to be *k*_−2_ = 0.01 *s* ^−1^, $k_{-2}^{\prime }=0.001\;\mu m^{2}s^{-1}$, *k*_−3_ = 0.04 *s* ^−1^ and *k*_−4_ = 10^−5^ *μm* ^4^ *s* ^−1^. Other parameters are given in the main text.(EPS)

S3 FigMolecular numbers (A-E) and total flux (F) as functions of *γ* which is changed *via* changing *k*_−4_.Only the cooperative-attachment model (solid curves) is able to oscillate (circles mark the bifurcation point). The parameters for cooperative model are chosen to be: *k*_−1_ = 0.06 *s* ^−1^, *k*_−2_ = 0.01 *s* ^−1^, $k_{-2}^{\prime }=0.001\;\mu m^{2}s^{-1}$ and *k*_−3_ = 0.04 *s* ^−1^. Other parameters are given in the main text. The non-cooperative model (without $k_{+2}^{\prime }$ and $k_{-2}^{\prime }$, dashed curves) has the same parameter values as the cooperative model except that *k*_+2_ = 1 *μms* ^−1^ and *k*_−2_ = 0.1 *s* ^−1^ to keep the molecular numbers and fluxes of the two models comparable.(EPS)

S4 FigDissipation rate (A), total flux (B) and cytoplasmic MinE number(C) as functions of *γ* which is changed by changing *k*_−4_.Oscillation occurs when *γ* enters the logarithmic region and 0.54 *s* ^−1^ < *k*_+4_ < 3.34 *s* ^−1^ (between cyan and blue curves). The dissipation rate in the oscillation region is close to the theoretical limit (dashed lines), especially when *k*_+4_ is small. (B) At relatively small *k*_+4_, average flux ⟨*J*⟩ increases monotonically with *γ*. However, when *k*_+4_ is large, the dependence can be non-monotonic (red curve). (C) Most MinEs are sequestrated on the membrane when *k*_+4_ < 3.34 *s* ^−1^. The parameters are chosen to be: *k*_−1_ = 0.06, *k*_−2_ = 0.01 *s* ^−1^, $k_{-2}^{\prime }=0.001\;\mu m^{2}s^{-1}$ and *k*_−3_ = 0.04 *s* ^−1^. Other parameters are given in the main text. For the no-cooperative model (without $k_{+2}^{\prime }$ and $k_{-2}^{\prime }$, dashed black curves), *k*_+2_ = 1 *μms* ^−1^, *k*_−2_ = 0.1 *s* ^−1^ and *k*_+4_ = 7 *s* ^−1^. Other parameters are the same as for the cooperative model.(EPS)

S5 Fig Time-averaged concentration of membrane-associated MinD along the cell’s long axis. *γ* is changed *via* changing *k*_−1_ (A, B), and *via* changing *k*_−4_ (C, D). Panels A, B (C, D) correspond to the solid and dashed curves in Fig 3B (D), respectively.
(EPS)

S6 FigCell-end effect in stable steady state.(A) In stable state, the membrane-associated MinD concentration can be slightly higher at mid-cell than poles, resulting in a negative performance. This is because the two ends of the cell absorb MinD:ATP and leave fewer MinD:ATP to the nearby membrane. (B) If we forbid MinD and MinE to bind the cell ends, the molecules are always evenly distributed along the cell’s long axis in stable steady state and therefore the performance is always *zero*. Parameters are chosen to be: *k*_−1_ = 30 *s* ^−1^, *k*_−2_ = 0.01 *s* ^−1^, $k_{-2}^{\prime }=0.001\;\mu m^{2}s^{-1}$, *k*_−3_ = 0.04 *s* ^−1^ and *k*_−4_ = 10^−5^ *μm* ^4^ *s* ^−1^. Other parameters are given in the main text.(EPS)

S7 Fig Contour plots of period in (*k*_−1_, *k*_+1_) (A), (*k*_−4_, *k*_+4_) (B) and (Δ*G*_1_,Δ*G*_4_) spaces at different Δ*G*_3_ (C), respectively. The parameters for A, B and C are the same as in Figs 2A, 2C and 4B in the main text, respectively. Under these parameters, the oscillation period is around 40s when *k*_+4_ ≈ 1 *s* ^−1^ and *k*_−4_ stays small.
(EPS)

S8 Fig Illustration of the definition of the performance. The cell length is normalized to be 1. The average concentration of MinD on the membrane is rescaled by the value at mid-cell. The performance is defined as h/w, which can be either positive (A) or negative (B).
(EPS)

S1 Movie Oscillatory dynamics of the MinCDE system. The five panels (from upper to lower) show the concentrations of MinD:ATP and MinE:MinD:ATP complexes on membrane, the concentrations of MinD:ADP, MinD:ATP, total MinD and MinE dimers in cytoplasm, respectively. This movie shows a canalized transfer of MinD molecules through the cytoplasm between the two poles.
(GIF)

S1 Text Additional discussion about dissipation rate, molecular numbers, flux, and reversibility. (PDF)
