# Supplementary figures and images for: An Optimal Free Energy Dissipation Strategy of the MinCDE Oscillator in Regulating Symmetric Bacterial Cell Division
Source: PLoS Comput Biol. 2015 Aug 28;11(8):e1004351. doi: 10.1371/journal.pcbi.1004351 (PMC4552557; doi:10.1371/journal.pcbi.1004351)

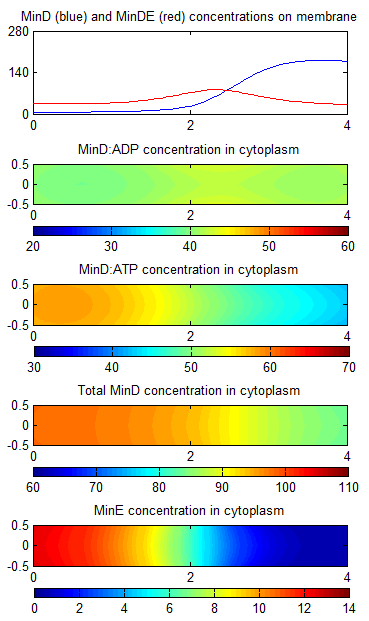

Supplement: S1 Movie — The five panels (from upper to lower) show the concentrations of MinD:ATP and MinE:MinD:ATP complexes on membrane, the concentrations of MinD:ADP, MinD:ATP, total MinD and MinE dimers in cytoplasm, respectively. This movie shows a canalized transfer of MinD molecules through the cytoplasm between the two poles. (GIF) [file pcbi.1004351.s009.gif]
