# Supplementary material for: An Optimal Free Energy Dissipation Strategy of the MinCDE Oscillator in Regulating Symmetric Bacterial Cell Division
Source: PLoS Comput Biol. 2015 Aug 28;11(8):e1004351. doi: 10.1371/journal.pcbi.1004351 (PMC4552557; doi:10.1371/journal.pcbi.1004351)
Supplement: S1 Text — (PDF) [file pcbi.1004351.s010.pdf]

Supporting Text S1: An optimal free-energy dissipation strategy of the MinCDE oscillator in regulating symmetric bacterial cell division

Liping Xiong, Ganhui Lan*,
Department of Physics, The George Washington University, Washington, DC 20052, USA
* E-mail: glan@gwu.edu

1 The dissipation rate, molecular numbers and flux as functions of γ

To understand how the dissipation rate of the MinCDE system changes with γ, we investigate different molecular numbers and flux as functions of γ here. Considering that the nucleotide exchange and MinE-aided MinD release steps are the two interfaces between the oscillator and the intracellular environment, we focus on changing γ *via* varying k_{-1} and k_{-4}.

1.1 γ is changed by changing k_{-1}

The MinCDE system shows a threshold (ultrasensitive-like) behavior as shown in Figs. S1 and S2. It is worth noting that this threshold behavior is not due to the cooperative attachment of MinD to the membrane because the threshold behavior still occurs after we remove the cooperative attachment (dashed curves in Fig. S1), despite that the sharpness of the curve (i.e. ultrasensitivity) is slightly reduced. In fact, the threshold behavior is a common feature of "futile cycles" where molecules are converted into each other with the total molecular number fixed. If all the reactions are irreversible, MinD:ADP is converted to MinD:ATP *via* reaction step 1, and MinD:ATP is converted back to MinD:ADP through reaction steps 2, 3 and 4. Including the backward reactions effectively reduces the "net" forward reaction rates to some extent. As shown in Fig. S1, with increasing γ (i.e., decreasing k_{-1}), the "net" forward reaction rate increases, resulting in more cytoplasmic MinD:ATP and membrane-associated MinD and MinE, as well as larger average flux, in a threshold manner.

However, k_{+1} sets the upper limit of the "net" forward reaction rate. If k_{+1} is not big enough, as $\gamma \to \infty$ (i.e., $k_{-1} \to 0$), the system will be saturated at a state with only a few MinD and MinE molecules bound on the membrane (cyan and yellow curves in Fig. S2). The large number of diffusive MinEs in cytoplasm prevent the accumulation of MinD on any site of the membrane, and therefore prevent oscillation from emerging. When k_{+1} is large enough ($> 0.89\ s^{-1}$), a large fraction of MinDs will bind to the membrane, recruiting and trapping nearly all the MinEs to the membrane at large γ (purple and blue curves in Fig. S2. The localization of the MinEs to the membrane paves the way for the oscillation. This is also why $N_E < N_D$ is a necessary condition for oscillation to occur [31].

An interesting observation is that the number of cytoplasmic MinD:ATP changes non-monotonically with γ (solid curve in Fig. S1B). This behavior only occurs when the cooperative attachment is included in the model. This is because cooperative attachment enhances the production rate of N_d from $N_{D:T}$, which only occurs when N_d & N_{de} are accumulated to a high level. Once the desired high level is reached, $N_{D:T}$ is rapidly consumed, causing a dramatic drop (Fig. S1B). This phenomena doesn't exist without the cooperative effect.

1.2 γ is changed by changing k_{-4}

The threshold behavior also exists when γ is changed by varying k_{-4} as shown in Fig. S3, although the γ dependence is smoother.

We have shown in the main text and Fig. S2A that the dissipation rate is a logarithmic function of γ in the oscillation region when γ is changed by changing k_{-1}. Here, we show in Fig. S4A that this is also true if γ is changed by changing k_{-4}. Therefore, we conclude that the logarithmic relation in oscillation region is a general behavior, independent of how γ is changed. This is because, as discussed in the main text, to generate oscillation, most MinEs should be sequestrated on the membrane [31], which results in a flux closing to the upper limit $k_{+4}N_E$. The dissipation rate can be therefore approximated by $k_{+4}N_E \ln \gamma$ (Eq. 2 in the main text).

Interestingly, when k_{+4} is large, the dissipation rate (Fig. S4A, red curve) and the average flux (Fig. S4B, red curve) show non-monotonic behavior. With increasing γ (i.e., decreasing k_{-4}), the flux tends to increase. However, the number of MinE on membrane decreases (Fig. S4C, red curve) which potentially decreases the flux. Therefore, at intermediate γ values, the competition between these two trends results in the non-monotonic behavior. Similar non-monotonic behavior is also observed in the dissipation rate and the

average flux with changing k_{+4} while all the other parameters are fixed. These non-monotonic behaviors are also attributed to the cooperative attachment, as in the model without cooperative attachment (Fig. S4A, B, dashed black curves), we do not see the non-monotonic behavior.

2 The importance of keeping the reactions reversible

The importance of keeping the reactions reversible is beyond the fact that no reaction is completely irreversible. Studying biological systems using reversible reaction models not only allows us to abstract the thermodynamic properties of the systems, but also reserves the possibility to capture the rich operational behaviors of the dynamic system.

Fig. 3C in the main text shows that for a model with irreversible reaction 4 (see $k_{-4} = 10^{-6}$ $\mu m^4 s^{-1}$ ($\gamma > 10^8$) for reference), oscillation only occurs at median k_{+4}, but not at small and large k_{+4}. If the effect of the backward reaction is just reduce the "net" forward reaction rate, one can expect that the system should also oscillate at large k_{+4} and k_{-4}. However, the fact is that once k_{+4} is larger than a threshold (about 3.34 s^{-1}), no oscillation occurs regardless of the k_{-4} value. This means that to generate oscillation the MinDE complex should be localized on the membrane for a while by a relatively "small" (but not too small) releasing rate k_{+4}, and this localization can not be realized by a relatively "large" backward rate k_{-4} (because the substrates for k_{-4}, MinD:ADP and MinE, are diffusing in the cytoplasm, and a large k_{-4} will in fact reduce the canalization effect as discussed in the main text). These qualitative and quantitative observations suggest that the forward and backward reactions often have different influences on the systems and should not be replaced by each other. To fully appreciate the control mechanism, reversible analysis is sometimes required.
